# Effect and Mechanism of LRP6 on Cardiac Myocyte Ferroptosis in Myocardial Infarction

**DOI:** 10.1155/2021/8963987

**Published:** 2021-10-19

**Authors:** Rui-lin Li, Cheng-hui Fan, Shi-yu Gong, Sheng Kang

**Affiliations:** ^1^Department of Cardiology, Shanghai East Hospital, School of Medicine, Tongji University, Jimo Road 150, Shanghai 200120, China; ^2^School of Medicine, Tongji University, Siping Road 1239, Shanghai 200092, China

## Abstract

**Background:**

This study was aimed at exploring the biological function and molecular mechanism of ferroptosis of LRP6 modulation in cardiomyocytes of myocardial infarction (MI).

**Method:**

We established the ferroptosis model of MI in vivo and in vitro and constructed the modulation network of circRNA-miRNA-LRP6 by bioinformatics analysis; then, we focused on exploring the regulatory relationship of LRP6 and its upstream genes circRNA1615 and miR-152-3p in the RIP experiments and the double luciferase reporter gene assay. Also, we tested the LRP6-mediated autophagy-related ferroptosis in MI.

**Results:**

Ferroptosis was found in cardiomyocytes of MI, and ferroptosis inhibitor Ferrostatin-1 (Fer-1) could improve the pathological process of MI. LRP6 was involved in the process of ferroptosis in cardiomyocytes, and LRP6 deletion regulated ferroptosis in cardiomyocytes through autophagy. Screening and identification of the upstream gene circRNA1615 would target LRP6. circRNA1615 inhibited ferroptosis in cardiomyocytes, and circRNA1615 could regulate the expression of LRP6 through sponge adsorption of miR-152-3p, prevent LRP6-mediated autophagy-related ferroptosis in cardiomyocytes, and finally control the pathological process of MI.

**Conclusions:**

circRNA1615 inhibits ferroptosis via modulation of autophagy by the miRNA152-3p/LRP6 molecular axis in cardiomyocytes of myocardial infarction.

## 1. Introduction

Myocardial infarction (MI) is the main cause of high incidence and sudden cardiac death; as the reperfusion follows, the ischemic myocardium aggravates its structural injuries, increases cardiomyocyte deaths, and expands the infarcted sizes, which further impairs cardiac function [1]. Despite that the mechanism of ischemic reperfusion (I/R) injury is not fully clarified, at present, the traditional explanations include the theories of calcium overload, leukocyte infiltration, and the massive release of reactive oxygen species. [2] However, in recent years, several researches reported new findings in the field. For example, autophagy limits, rather than exacerbates, myocardial damage on infarction. Nevertheless, abnormal mitophagy is maladaptive and has been linked to cell death [3]. Mitochondrial reactive oxygen species (mROS) drive both acute emergent events, such as electrical instability responsible for sudden cardiac death, and those that mediate chronic heart failure remodeling, characterized by suppression or altered phosphorylation of metabolic, antioxidant, and ion transport protein networks [4]. In the I/R injuries of mouse heart, the iron chelators and glutaminolysis inhibitors significantly mitigated cardiomyocyte cell death and damage of heart tissue and function [5]. Thus, it is crucial to solve the myocardial injury effectively for the better therapy of MI and its prognosis.

The toxicity of iron and lipid peroxidation were reported in the 1900s and 1950s, respectively. As an evolutionary conservative program, ferroptosis plays a vital role in the development and diseases of various organisms [6].

The transcription factor BTB domain and CNC homolog 1 (BACH1), a regulator in heme and iron metabolism, represses the genes that combat labile iron-induced oxidative stress, and controls the threshold of ferroptosis induction, then alleviates ferroptosis-related heart damage in MI [7]. At the genetic level, the activation of the rapamycin kinase (MTOR) or ectonucleotide pyrophosphatase/phosphodiesterase 2 (ENPP2) pathway inhibits ferroptosis-mediated cardiomyocyte injury [8]. Thus, ferroptosis mediates acute myocardial injury. However, it is not clear about the effect of ferroptosis and its molecular mechanism in the process of MI.

Low-density lipoprotein receptor-related protein 6 (LRP6) is involved in the process of cardiomyopathy. LRP6 deletion promotes autophagy [9]. Autophagy is a necessary factor in ferroptosis [10], and the autophagy agonist rapamycin was loaded into mouse cardiomyocytes to increase the sensitivity of ferroptosis, indicating that LRP6 may be closely related to autophagy and ferroptosis of cardiomyocytes [11].

Autophagy-related cyclic RNAs (ACR circRNAs) attenuate cardiomyocyte autophagy and cell death by regulating the phosphatase and tensin homolog-induced putative kinase1 (Pink1)/family with the sequence similarity 65 member B (FAM65B) pathway, protect the heart from I/R injury, and reduce MI size [12]. All these suggested that circRNAs might be involved in cardiac repair.

The study was aimed at exploring the effect and mechanism of LRP6 on cardiac myocyte ferroptosis in MI, screening and identifying upstream genes circRNA targeting LRP6, and investigating the relationships of circRNA modulation through LRP6 and autophagy.

## 2. Methods

### 2.1. Animal Models

The animal experiments conformed to the Guide for the Care and Use of Laboratory Animals (US and National Institutes of Health). C57BL/6J male mice of SPF grade, 8-10 weeks old, were used. The day before operation, the mice in the MI+ferrostatin-1 (Fer-1) group were injected a dose of 1 mg/kg ferroptosis inhibitor Fer-1 (SML0583-5MG, Sigma-Aldrich, USA). Fer-1 was dissolved in dimethyl sulfoxide (DMSO), then diluted in sterile saline. The sham group and the MI+NS group were injected with the same dose of saline (NS). The mice were anesthetized by 3% pentobarbital sodium via intraperitoneal injection, and the MI model was established by ligating the anterior descending branch of the left coronary artery (LAD) for 30 min. The mice in the sham group were not ligated with LAD. We constructed the MI model and completed the sampling time points according to the report [13]. Because there were few ferroptosis-related studies, our experiment was designed according to a previous study, including the dosages of the ferroptosis inhibitor and time points [14]. Statistically, the death rate of model mice was approximately 20% during procedures.

### 2.2. Echocardiography Measurement

On the 7th day of MI, the left ventricular end-diastolic and end-systolic diameters (LVIDd and LVIDs) of mice were measured by the small animal ultrasound Shenzhen Mairui Biomedical DP-50ev (Shenzhen Mindray Bio-Medical Electronics Co., China), and the ejection fraction (EF%) and left ventricular short-axis shortening (FS%) were calculated using edge-detection software and standard techniques.

### 2.3. Histology

Midventricular short-axis heart sections (5 mm) from MI model hearts were fixed in 2% paraformaldehyde (PFA) overnight at 4°C, dehydrated in 70% ethyl alcohol, and embedded in paraffin. The sections were stained with H&E and examined under a light microscope.

### 2.4. Cell Culture and Intervention

The mouse cardiomyocytes (HL-1 cells, ZQ0920, Shanghai Zhong Qiao Xin Zhou Biotechnology, China) were cultured with DMEM (10-013-CVRC, Corning, USA), 10% fetal bovine serum (Gibco, Thermo Fisher Scientific, USA), and 1% PBS (E607011, Shanghai Sangon Biotech, China) at 95% air, 5% CO_2_, and 37°C.

The HL-1 cells were adjusted and made into a single-cell suspension. After counting, each group of cells was made into a 2 × 10^4^ cells/ml single-cell suspension. Cardiomyocytes were treated with 0, 2.5, 5, 10, 20, and 40 *μ*M erastin (HY-15763, MCE, USA), 10 *μ*M ZVAD-FMK (219007-250UG, MCE, USA), and 0.5 *μ*M necrosulfonamide (1360614-48, MCE, USA). After the cells were made into 1 × 10^4^ cell/ml, each well was covered with 100 *μ*l cells, that is, 1000 cells/well. There were 6 multiple wells in each sample, and the edge wells were added with 100 *μ*l phosphate-buffered solution (PBS), which were cultured at 37°C in a 5% CO_2_ incubator.

HL-1 cells were transfected with mimics/inhibitor of negative control (NC) and LRP6, and Autophagy-Related 5 (ATG5) was tested using the Lipofectamine™ 2000 Transfection Reagent Kit (Thermo Fisher Scientific, Waltham, MA, USA) according to the reverse transfection method of the manufacturer's protocol.

### 2.5. Cell Viability Assay

After experimental treatment conditions were finished, the cells were made into 1 × 10^4^ cell/ml, and each well was covered with 100 *μ*l cells. There were 6 multiple wells in each sample, and the edge wells were added with 100 *μ*l PBS. The cells were cultured in a hypoxia bag at 37°C in a 5% CO_2_ incubator. The cells were treated with CCK-8, and detected by an enzyme labeling instrument. Then, 10 *μ*l CCK-8 (Beyotime Biotechnology, China) was added into the well 48 hours later, and the liquid replacement was not required. After 1 hour, the OD value was detected by a 450 nm enzyme labeling instrument (Infinite M1000, Tecan, Switzerland).

### 2.6. Lipid Peroxidation Assay

Lipid peroxidation was determined by measuring the amount of malondialdehyde (MDA) using a Lipid Peroxidation Assay Kit (s0131, Beyotime Biotechnology, China) according to the manufacturer's instructions. Briefly, HL-1 cells were homogenized with lysis buffer, and the supernatant was prepared with a thiobarbituric acid- (TBA-) glacial acetic acid reagent. After incubation at 95°C for 1 h, the MDA TBA adduct was quantified colorimetrically at 532 nm using an enzyme labeling instrument (Infinite M1000, TECAN, Switzerland). After calculating the MDA content in the sample solution, the MDA content in the initial sample was expressed by the tissue weight per unit, *μ*mol/mg.

### 2.7. Detection of Iron by Colorimetry

The pretreatment of iron-containing DMEM-cultured HL-1 cells were described as the foregoing method. Iron was detected by the Iron Colorimetric Assay Kit (E1042, Beijing Applygen Technology, China) according to the manufacturer's instructions. The absorbance was determined by 550 nm, which drew the standard curve and calculated the concentration of iron.

### 2.8. Western Blot Analysis

Briefly, after collecting the supernatants of the cell lysates, they were heat-denatured (incubated 3 times at 99°C, each time for 5 min; fully shaken; and briefly centrifuged at 12,000 rpm for 3 min). 15 *μ*l of the denatured sample buffer was slowly added into the sample slot, and the protein samples were separated by sodium dodecyl sulfate polyacrylamide gel electrophoresis (SDS-PAGE) and transferred to polyvinylidene difluoride (PVDF) membranes. The membranes were incubated by the primary antibodies diluted overnight with 5% BSA for 4°C. The matching secondary antibodies were diluted with the blocking solution, and the PVDF membranes were incubated by the secondary antibodies for 1-2 hours at room temperature. A high sensitive ECL luminescence kit was used for color reaction. Further analysis was carried out using a chemiluminescence imaging analysis system (Shanghai Clinx Science Instruments Co., China) to quantify the protein bands.

The following blotting reagents and antibodies were used: a previous prepared cell lysate (RIPA cracking solution, Thermo Fisher Scientific, USA), a concentrated glue and separation glue (Thermo Fisher Scientific, USA), anti-GAPDH (60004-1-Lg, Proteintech, USA), anti-LRP6 (sc-25317, Santa Cruz Biotechnology Inc., USA), goat anti-mouse IgG HRP (ab205719 or ab6721, Abcam, Britain), anti-LC3-A/B (12741T, CST, USA), ATG5 (ab109490, Abcam, Britain), P62 (5114T, CST, USA), and a high sensitive ECL luminescence kit (Thermo Fisher Scientific, USA).

### 2.9. RNA Extraction, RT-PCR, and Sample Sequencing

Total RNA was isolated using TRIzol (Invitrogen, USA) according to the manufacturer's protocol. cDNA was synthesized from 1 *μ*g of total RNA using the reverse transcription kit (Thermo Scientific, #K1622, Thermo Fisher Scientific, USA) according to the manufacturer's protocol. The amplified cDNA was analysed using real-time PCR (ABI Q6, Applied Biosystems Inc., USA) with the following primers: GAPDH (forward), CAAAATGGTGAAGGTCGGTGT; GAPDH (reverse), GAGGTCAATGAAGGGGTCGTT; circ_0001615 (forward), ATGTTTCTGGAGCAGCAAGTGA; circ_0001615 (reverse), CGAGAAGCCTGTCAACTGAG; NC (forward), UUCUCCGAACGUGUCACGUTT; NC (reverse), ACGUGACACGUUCGGAGAATT; miR-152-3p (forward), GCAGTCAGTGCATGACAGA; miR-152-3p (reverse), AGTGCGTGTCGTGGAGTCG; LRP6 (forward), ACAAATATACTGGCGAGGGTCT; LRP6 (reverse), GGAGACATCAAACACAAATGGGA; ATG5 (forward), TGCGGTTGAGGCTCACTTTA; ATG5 (reverse), GTTGATGGCCCAAAACTGGT.

### 2.10. Double Luciferase Reporter Experiments

3′UTR sequences of circRNA1615 containing wild-type (WT) or mutant (MUT) binding sites of miR-152-3p were amplified and cloned into the PUC57 vector (target DNA fragment vector) to generate circRNA1615-WT or the circRNA1615-MUT Luciferase reporter vector, respectively. The transfection plasmids were the PsiCHECK-2 Vector (Promega, Madison, WI, USA). The HL-1 cells were cotransfected with circRNA1615-WT or circRNA1615-MUT and miR-152-3p mimics (miR-152-3p) or miR-152-3p negative control (miR-NC) according to the instructions of the manufacturer of Lipofectamine™ 2000 Transfection Reagent (Thermo Fisher Scientific, Waltham, MA, USA). The same was followed in the transfection of LRP6-WT or LRP6-MUT in the HL-1 cells. After 48 h transfection, the relative Luciferase activity was detected with the Dual-Luciferase Reporter Assay Kit (Promega, Madison, WI, USA). The firefly Luciferase was normalized to the Luciferase activity.

### 2.11. RNA-Binding Protein Immunoprecipitation (RIP) Assay

RIP assay was performed using the Magna RI RNA-Binding Protein Immunoprecipitation Kit (Millipore, Billerica, MA, USA) according to the manufacturer's instructions. The magnetic beads (Bio-Rad, Hercules, CA, USA) were incubated with Argonaute-2 antibody (Anti-Ago2) or Immunoglobulin G antibody (Anti-IgG). Then, the HL-1 cell extract was incubated with RIP buffer containing magnetic beads conjugated to human anti-AGO2 antibody (Millipore, Billerica, MA, USA) and IgG control (Millipore, Billerica, MA, USA). The coprecipitated RNA was purified and quantified by qRT-PCR. RNA integrity was assessed with an Agilent 2100 Bioanalyzer.

### 2.12. Statistical Analysis

Continuous data were expressed as the means ± standard error (SEM). Data were analyzed using GraphPad Prism (version 8.0), and the plots were generated by GraphPad Prism. The comparisons between two groups were calculated using the two-tailed *t*-test. For comparison across multiple experimental groups, one-way analysis of variance (ANOVA) was used, followed by Bonferroni's post hoc test. Statistical significance was set at *P* < 0.05.

## 3. Results

### 3.1. Ferroptosis Inhibitor Improved the Pathological Process of MI

**Figure 1:**
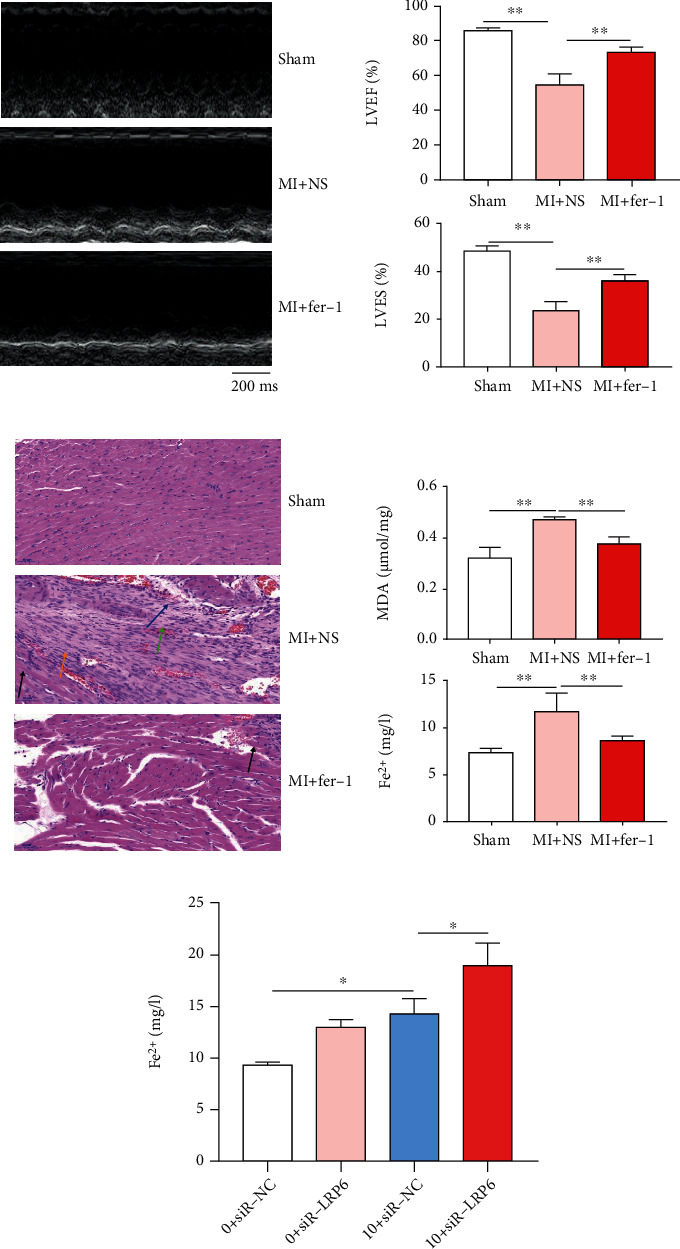
Ferroptosis inhibitor improves the pathological process of MI. (a, b) The LVEF% and LVFS% in both the MI group and the control group. Time stamp: 200 ms; scale bar: 1 mm. Pooled data: biological replication indicated five mice in each group. Data were means ± s.e.m.^∗∗^*P* < 0.01 compared with ctrl by one-way analysis of variance (ANOVA) with Bonferroni's post hoc test. (c) Cardiomyocytes' death were stained with H&E in the sham group, the MI+NS group, and the MI+Fer-1 group. Scale bar: 50 *μ*m. The presented images were representative. (d) Evaluation of MDA and Fe^2+^ levels in infarcted myocardial tissue. Upper: MDA level; bottom: Fe^2+^ level. Pooled data: biological replication indicated five mice in each group. (e) Effect of Fer-1 mediated the viability of cardiomyocytes treated with erastin, ZVAD-FMK, or necrosulfonamide. Pooled data: biological replication indicated five mice in each group. Data were means ± s.e.m.^∗^*P* < 0.05 and ^∗∗^*P* < 0.01 compared with ctrl by one-way ANOVA with Bonferroni's post hoc test.

The LVEF% and LVFS% in the MI group were significantly lower than those in the control group (Figures 1(a) and 1(b)). Further, we added more exact details of the ultrasonograph in supplementary Figure 1. The cardiomyocytes were closely arranged, and their morphology was natural in the sham group; however, in the MI group, the injured myocardial cells presented with coagulative necrosis (black arrow), disappearing cardiomyocytes with new granulation tissue and gradual fibrosis (blue arrow), inflammatory cell infiltration (orange arrow), and bleeding (green arrow) (Figure 1(c)). Further, the MI group had higher levels of MDA and Fe^2+^ in the infarcted myocardial tissue (Figure 1(d)). The ferroptosis inhibitor reversed the low LVEF% and LVFS% in infarcted mice (Figures 1(a) and 1(b)), which showed mild cell injury and a few coagulative necrosis of cardiomyocytes (black arrow) (Figure 1(c)), and decreased the levels of MDA and Fe^2+^ in infarcted myocardial tissue (Figure 1(d)). Noticeably, Fer-1 significantly improved the viability of cardiomyocytes treated with erastin (ferroptosis inducer), but ZVAD-FMK (apoptosis inhibitor) and necrosulfonamide (necrosis inhibitor) did not have similar effects (Figure 1(e)).

Thus, in the established mouse MI model, we found that ferroptosis occurred in mouse MI. Further, ZVAD-FMK (apoptosis inhibitor) and necrosulfonamide (necrosis inhibitor) did not improve the viability of cardiomyocytes treated with erastin (ferroptosis inducer), but Fer-1 (ferroptosis inhibitor) could improve left ventricular function and pathological changes of MI.

### 3.2. LRP6 Was Involved in the Process of Ferroptosis in Cardiomyocytes through Autophagy

**Figure 2:**
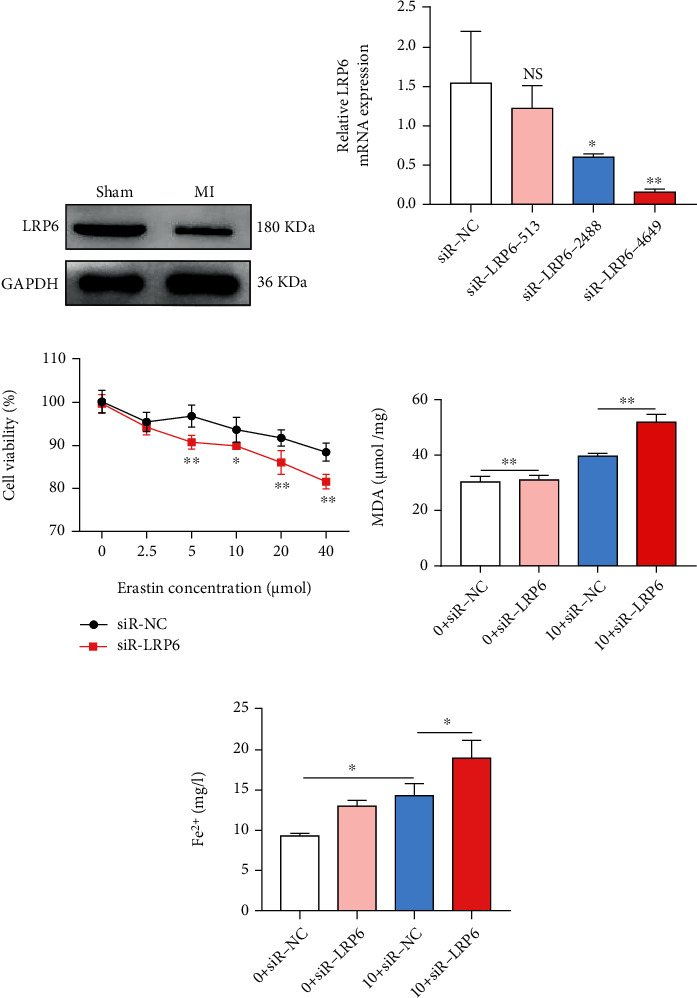
LRP6 deletion promotes ferroptosis in cardiomyocytes. (a) The protein expression of LRP6 in the infarcted myocardial tissue was detected using western blot analysis with SDS-PAGE. Top: typical blots; bottom: pooled data, biological replication indicated five mice in each group. Data were means ± s.e.m. (b) Analysis of expression of LRP6 transfected with LRP6-siR-mus-4649, LRP6-siR-mus-2488, or LRP6-siR-mus-513. Pooled data: biological replication indicated five mice in each group. Data were means ± s.e.m.^∗^*P* < 0.05 and ^∗∗^*P* < 0.01 compared with ctrl by one-way ANOVA with Bonferroni's post hoc test. (c) Evaluation of the activity of cardiomyocytes interfered with LRP6 following induction of hypoxia and erastin. Pooled data: biological replication indicated five mice in each group. Data were means ± s.e.m.^∗^*P* < 0.05 and ^∗∗^*P* < 0.01 compared with ctrl by two-tailed unpaired Student's *t*-test. (d, e) Representative of the levels of MDA and Fe^2+^ in cardiomyocytes following induction of hypoxia and erastin in 48 hours. 0 and 10 indicated the concentrations of ferroptosis inducer erastin (*μ*M), respectively. Pooled data: biological replication indicated five mice in each group. Data were means ± s.e.m.^∗^*P* < 0.05 and ^∗∗^*P* < 0.01 compared with ctrl by one-way ANOVA with Bonferroni's post hoc test.

The expression of LRP6 in the infarcted myocardial tissue was significantly lower when compared to the control group (Figure 2(a)). LRP6-related interference fragment siRNA was transfected into cardiomyocytes, and the expression of LRP6 in cardiomyocytes was significantly downregulated after transfection with LRP6-siR-mus-4649 compared with LRP6-siR-mus-2488 or LRP6-siR-mus-513 (Figure 2(b)). After induction of hypoxia and erastin, compared with the control group, the activity of cardiomyocytes interfered by LRP6 was lower (Figure 2(c)), and the levels of MDA and Fe^2+^ in cardiomyocytes were higher (Figures 2(d) and 2(e)).

**Figure 3:**
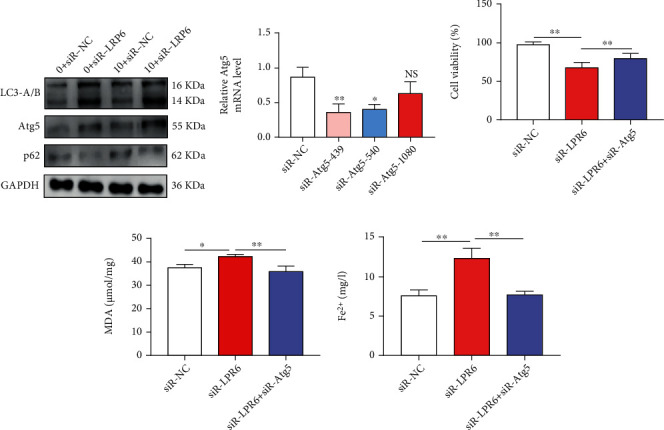
LRP6 deletion regulates ferroptosis in cardiomyocytes through autophagy. (a) The presented blots were obtained from the expression of autophagy-related proteins LC3-A/B, ATG, and p62 after LRP6 interference in the cardiomyocytes treated with hypoxia and erastin. Top: typical blots. Pooled data: biological replication indicated five mice in each group. (b) Effect of ATG5 siRNA mediated the expression of ATG5 in cardiomyocytes. Pooled data: biological replication indicated five mice in each group. Data were means ± s.e.m.^∗^*P* < 0.05 and ^∗∗^*P* < 0.01 compared with ctrl by one-way ANOVA with Bonferroni's post hoc test. (c) Analysis of the cardiomyocytes with LRP6 siRNA and ATG5 siRNA influence on survival rate in cardiomyocytes treated with hypoxia and erastin. Statistical analysis was obtained from five mice in each group. (d, e) In this case, the presented Fe^2+^ and MDA levels were induced by LRP6 deletion. Pooled data: biological replication indicated five mice in each group. Data were means ± s.e.m.^∗^*P* < 0.05 and ^∗∗^*P* < 0.01 compared with ctrl or si-LRP6 by one-way ANOVA with Bonferroni's post hoc test.

In the cardiomyocytes treated with hypoxia and erastin, the expression of autophagy-related proteins LC3-A/B (microtubule associated protein 1 light chain 3-A/B) and ATG5 after LRP6 interference was higher when compared with the control group, but the expression of sequestosome-1 (p62) was lower (Figure 3(a)). ATG5 siRNA significantly decreased the expression of ATG5 in cardiomyocytes (Figure 3(b)). In cardiomyocytes treated with hypoxia and erastin, the cardiomyocytes with LRP6 siRNA and ATG5 siRNA could repair the decreasing survival rate induced by LRP6 deletion (Figure 3(c)) and reverse the increase of Fe^2+^ and MDA induced by LRP6 deletion (Figures 3(d) and 3(e)).

Thus, to explore whether LRP6 regulates ferroptosis through autophagy, we detected the expression of LRP6 in myocardial tissue and found the low expression of LRP6 in the MI group; interference with LRP6 could induce ferroptosis of cardiomyocytes, promote the expression of autophagy-related proteins LC3-A/B and ATG5, and reduce the expression of p62. In addition, the cell was interfered with LRP6 and inhibited autophagy simultaneously, its survival rate rose, and the level of ferroptosis decreased.

### 3.3. Screening and Identification of circRNA1615 Plays a Role as a miRNA Sponge by Targeting LRP6

**Figure 4:**
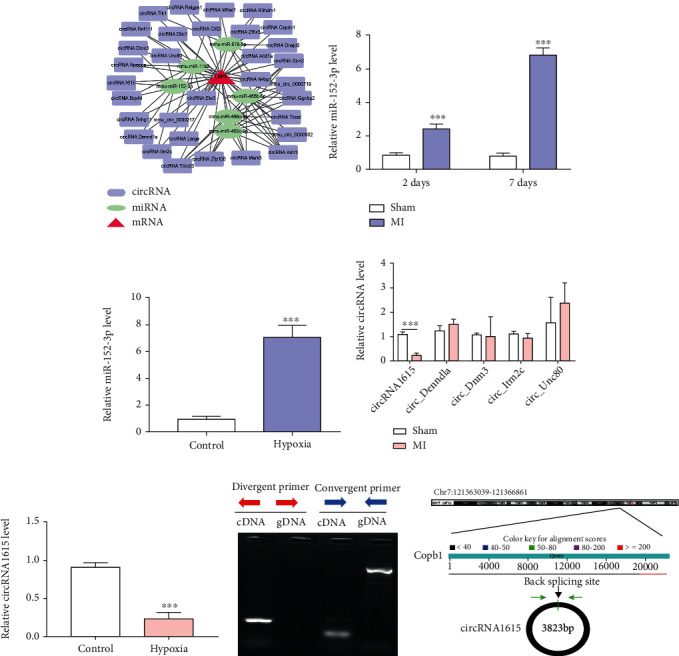
Screening and identification of circRNA1615 targeting LRP6 in the process of MI. (a) To construct a circRNA-miRNA-LRP6 regulatory network through the CLIP database. (b and c) Based on the analysis of LRP6 regulatory network. Evaluation of the binding ability of mmu-miR-466c-5p, mmu-miR-466o-5p, mmu-miR-679-5p miRNA, and the representative of the expression of miR-152-3p in the GEO database data (GSE90123) and our experiment. (d) The expression of five circRNAs targeting miR-152-3p in mouse myocardial tissue. (e) Effect of the expression of circRNA1615 after hypoxia. Pooled data: biological replication indicated five mice of each group in (b–e). Data were means ± s.e.m. ^∗∗∗^*P* < 0.001 compared with ctrl by two-tailed unpaired Student's *t*-test. (f and g) Representative of circRNA1615 derived from Copb1 gene.

We screened the miRNA targeting LRP6 and the circRNAs that bound to these miRNAs through the CLIP database to construct a circRNA-miRNA-LRP6 regulatory network (Figure 4(a)). Based on the analysis of the LRP6 regulatory network, it was found that mmu-miR-466c-5p, mmu-miR-466o-5p, and mmu-miR-679-5p miRNA had strong binding abilities, while the GEO database data (GSE90123) showed that the expression of miR-152-3p was enriched in exosomes derived from H9c2 cells under hypoxic conditions in 4 hours [15], and we also found that it significantly rose at 7 days in the hypoxia-treated cardiomyocytes (Figures 4(b) and 4(c)). The expression of five circRNAs targeting miR-152-3p was detected in mouse myocardial tissue, and the expression of circRNA1615 was significantly downregulated in the MI group (Figure 4(d)). Further, the expression of circRNA1615 reduced significantly after hypoxia (Figure 4(e)). Using polymeric primers and divergent primers to amplify gDNA and cDNA of mouse myocardial tissue, only divergent primers amplified circRNA1615 (circBase: mmu_circ_0001615) in cDNA. The details of the identification of circRNA1615 could be traced back to the circbase webstation (http://www.circbase.org/cgi-bin/singlerecord.cgi?id=mmu_circ_0001615) and supplementary Figure 2. Importantly, the sequencing of PCR products revealed that circRNA1615 was a circular RNA derived from the Copb1 gene (Figures 4(f) and 4(g)).

**Figure 5:**
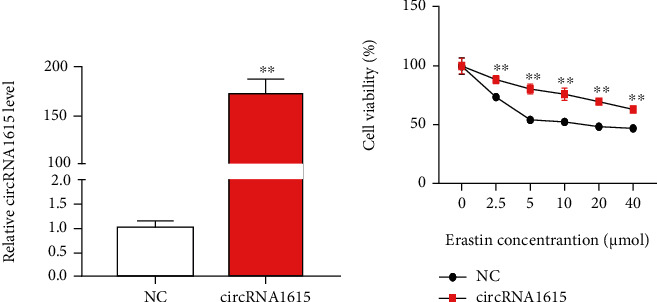
circRNA1615 inhibits ferroptosis in cardiomyocytes. (a) The presented expression of circRNA1615 in mouse cardiomyocytes transfected with overexpressed plasmids. (b) Analysis of the survival rate of overexpressed circRNA cells treated with hypoxia and erastin. Pooled data: biological replication indicated five mice in each group. Data were means ± s.e.m. ^∗∗^*P* < 0.01 compared with ctrl by two-tailed unpaired Student's *t*-test.

We constructed the overexpressed circRNA1615 plasmids, then transfected them to cardiomyocytes. It was found that the expression of circRNA1615 in mouse cardiomyocytes transfected with overexpressed plasmids was higher than that of control plasmids (Figure 5(a)). The survival rate of overexpressed circRNA cells increased in cardiomyocytes treated with hypoxia and erastin (Figure 5(b)), suggesting that circRNA1615 inhibits ferroptosis in cardiomyocytes.

Thus, to study whether the circRNAs are involved in the regulation of ferroptosis by LRP6, we constructed a circRNA-miRNA-LRP6 regulatory network. Bioinformatics analysis combined with experimental tests showed that the expression of miR-152-3p in myocardial tissue of infarcted mice and hypoxia-treated cardiomyocytes was increased, while the matching circRNA1615 expression was downregulated. Noticeably, the overexpression of circRNA1615 could inhibit cardiomyocyte death (Figures 4 and 5).

**Figure 6:**
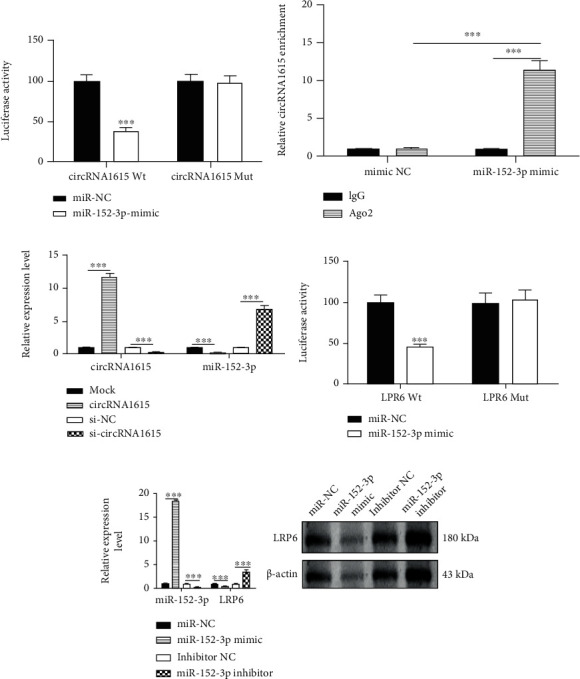
circRNA1615 targets LRP6 through sponge miR-152-3p. (a) Luciferase assay for the ability of circRNA1615 binding to miR-152-3p. (b) circRNA1615 precipitated with miR-152-3p and AGO2 antibodies in an RIP experiment. (c) The expression of miR-152-3p in cardiomyocytes after overexpression or knocking down of circRNA1615. (d) Analysis of the binding sites of miR-152-3p and LRP6. (e) The presented expression of LRP6 after overexpression or knocking down of miR-152-3p. Left: pooled data. Right: the presented typical blots were representative. Pooled data (a–e): biological replication indicated five mice in each group. Data were means ± s.e.m.^∗∗∗^*P* < 0.001 compared with ctrl by one-way ANOVA with Bonferroni's post hoc test.

In addition, we studied whether circRNA1615 has the ability to bind to miRNA. The binding sites of circRNA1615 and miR-152-3p were analyzed in the database, and we used the software RNAhybrid (version: 2.2) [16] to predict the binding sites of circRNA1615 and miR-152-3p, miR-152-3p, and LRP6 (Supplementary Figure 3). Further, the psicheck-2 reporter gene wild-type vector (including miR-152-3p target sequence) and the mutant vector (miR-152-3p target sequence point mutation) of LRP6 were constructed and cotransfected with miR-152-3p mimics. Compared with the negative control RNA (NC), miR-152-3p decreased the luciferase activity of the wild-type vector (Figure 6(a)); the RIP experiment showed that circRNA1615 could be precipitated by miR-152-3p and AGO2 antibodies, indicating the sponge adsorption ability of circRNA1615 to miR-152-3p (Figure 6(b)). The expression of miR-152-3p in cardiomyocytes was diminished after overexpression of circRNA1615, while the expression of miR-152-3p was upregulated by knocking down circRNA1615 (Figure 6(c)). To test whether miR-152-3p targets the binding sites of miR-152-3p and LRP6 in the database, the wild-type psicheck-2 reporter gene vector and mutant vector of LRP6 were constructed and cotransfected with miR-152-3p mimics and miR-NC. Compared with the negative control RNA (NC), miR-152-3p decreased the luciferase activity of the wild-type vector (Figure 6(d)). Overexpression of miR-152-3p in cardiomyocytes inhibited the expression of LRP6, while knocking down miR-152-3p upregulated the expression of LRP6 (Figure 6(e)).

Therefore, the overexpression or knockdown of circRNA1615 in cardiomyocytes negatively regulated the expression of miR-152-3p, and the luciferase and RIP experiments verified the spongy adsorption of circRNA1615 to miR-152-3p. In addition, the overexpression or knockdown of miR-152-3p in cardiomyocytes could negatively regulate the expression of LRP6, and the luciferase reporter gene assay confirmed the targeting effect of miR-152-3p on LRP6 (Figure 6). These results suggest that circRNA1615 regulates the expression of LRP6 through sponge adsorption of miR-152-3p, and then it modulates ferroptosis in cardiomyocytes.

## 4. Discussion

**Figure 7:**
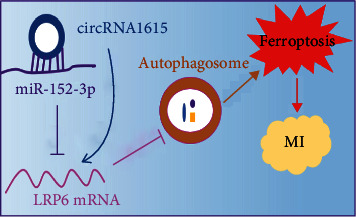
The diagram of the mechanism hypothesis. circRNA1615 may control the spongy adsorption of miR-152-3p targeting LRP6, then inhibit LRP6-mediated autophagy-related ferroptosis in cardiomyocytes, and finally regulate the pathological process of MI.

At present, the effect and molecular mechanism of ferroptosis in the pathological development of MI are not clear. We explored the effect and molecular mechanism of LPR6 on cardiomyocyte ferroptosis by establishing a model of MI in vivo and in vitro, constructed the regulatory network of circRNA-miRNA-LRP6 by bioinformatics analysis, and tested the molecular mechanisms of circRNA1615 regulating ferroptosis in the RIP experiment and using the double luciferase reporter gene assay. Based on our findings, circRNA1615 modulated the expression of LRP6 through its sponge adsorption of miR-152-3p, and then adjusted for the LRP6-mediated autophagy-related ferroptosis in cardiomyocytes of MI (Figure 7).

In the reperfusion injury caused by heart transplantation or coronary occlusion, cardiomyocytes will incur ferroptosis and release inflammatory mediators to aggravate the myocardial injury. Ferroptosis inhibitor Fer-1 reduced cardiomyocyte death and prevented the recruitment of neutrophils, diminished the infarct size, and improved left ventricular systolic function and left ventricular remodeling [17]. Quantitative proteomic analysis shows that the downregulation of glutathione peroxidase 4 (Gpx4) in MI contributes to the ferroptosis of cardiomyocytes [18], and clinical studies have shown that myocardial iron is a risk factor for left ventricular remodeling after MI [19]. In our study, the ferroptosis inhibitor reversed the low LVEF% and LVFS% in infarcted mice (Figures 1(a) and 1(b)), showed the mild cell injury and a few coagulative necrosis of cardiomyocytes (black arrow) (Figure 1(c)), and decreased the levels of MDA and Fe^2+^ in infarcted myocardial tissue (Figure 1(d)). Moreover, Fer-1 significantly improved the viability of cardiomyocytes treated with erastin (ferroptosis inducer), but ZVAD-FMK (apoptosis inhibitor) and necrosulfonamide (necrosis inhibitor) did not have similar effects (Figure 1(e)), suggesting that ferroptosis might be a novel therapeutic target for MI.

LRP6 focuses on lipid homeostasis and glucose metabolism associated with the rapamycin target protein (mTOR) pathway [20]. Targeting LRP6-mediated signaling pathways can reduce neointimal formation and myocardial I/R injury [21]. Further, cardiac LRP6 deletion inhibited autophagy degradation and fatty acid utilization, and together with the activation of dynamin-related protein1 (Drp1) and downregulation of nuclear transcription factor EB (TFEB), subsequently brought fatal dilated cardiomyopathy and cardiac dysfunction [22]. Importantly, the mitochondrial DNA stress triggers autophagy-dependent ferroptotic death [23]. ROS-mediated autophagy increases intracellular iron levels and ferroptosis by ferritin and transferrin receptor regulation [24].

In our study, the expression of LRP6 in the infarcted myocardial tissue was significantly lower in comparison to the control group (Figure 2(a)). LRP6-related interference fragment siRNA was transfected into cardiomyocytes, and the expression of LRP6 in cardiomyocytes was significantly downregulated after transfection with LRP6-siR-mus-4649 (Figure 2(b)). After induction of hypoxia and erastin, compared with the control group, the activity of cardiomyocytes interfered by LRP6 was lower (Figure 2(c)), and the levels of MDA and Fe^2+^ in cardiomyocytes were higher (Figures 2(d) and 2(e)). Thus, the interference with LRP6 could promote ferroptosis of cardiomyocytes. In addition, siR-LRP6 increased the expression of autophagy-related proteins LC3-A/B and ATG5, and decreased the expression of p62. Nevertheless, abnormal mitophagy is maladaptive and has been linked to cell death. Both LRP6 and autophagy were inhibited simultaneously, the cell survival rate increased, and the level of ferroptosis diminished (Figure 3). These results confirmed that LRP6 deletion could promote autophagy, then trigger ferroptosis of cardiomyocytes, and participate in the regulation of MI pathological process.

Unlike the traditionally known RNA species, the sequence of noncoding circRNA is not arranged in a normal order relative to the genomic context but arrays in a scrambled manner, in which the 3′ downstream sequences are joined to the 5′ upstream sequences, resulting in a covalent-closed circular molecule without free terminals. In addition, circular RNAs are abundant, conserved, and associated with ALU repeats [25]. circRNA Nfix (circNfix) deletion can induce myocardial regeneration after MI in adult mice [26]. Overexpression of circFndc3b (derived from exons 2 and 3 of the Fndc3b gene) in infarcted heart can reduce cardiomyocyte apoptosis, enhance neovascularization, and improve left ventricular function [27]. The expression of circNFIB (circBase: mmu_circ_0011794) in the mouse heart after MI is decreased, and the upregulation of circNFIB, can reduce myocardial fibrosis by inhibiting miR-433 [28].

In our study, we constructed a circRNA-miRNA-LRP6 regulatory network. Bioinformatics analysis combined with experimental tests showed that the expression of miR-152-3p in myocardial tissue of the infarcted mice and the hypoxia-treated cardiomyocytes was increased, while the matching circRNA1615 expression was downregulated. Noticeably, the overexpression of circRNA1615 could inhibit cardiomyocyte death (Figures 4 and 5). Further, the overexpression or knockdown of circRNA1615 in cardiomyocytes negatively regulated the expression of miR-152-3p as well as the spongy adsorption of circRNA1615 to miR-152-3p. In addition, the overexpression or knockdown of miR-152-3p in cardiomyocytes could negatively control the expression of LRP6, and the targeting effect of miR-152-3p on LRP6 (Figure 6), thereby our findings suggested that circRNA1615 would regulate the expression of LRP6 through its sponge adsorption of miR-152-3p.

We are glad and appreciate that our findings have been published as a preprint [29].

## 5. Conclusion

circRNA1615 inhibits ferroptosis via modulation of autophagy by the miRNA152-3p/LRP6 molecular axis in cardiomyocytes of myocardial infarction.

## Data Availability

The data used to support the findings of this study are available from the corresponding author upon request.

## Supplementary Materials

Supplementary Materials Supplementary Figure 1 The measurement of ultrasonograph in the representative mouse. Supplementary Figure 2. The detail of identification of circRNA1615 circbase database. Supplementary Figure 3. The binding sites of circRNA1615, miR-152-3p, and LRP6.
