# Supplementary material for: Effect and Mechanism of LRP6 on Cardiac Myocyte Ferroptosis in Myocardial Infarction
Source: Oxid Med Cell Longev. 2021 Oct 19;2021:8963987. doi: 10.1155/2021/8963987 (PMC8548150; doi:10.1155/2021/8963987)
Supplement: Supplementary Materials — Supplementary Figure 1 The measurement of ultrasonograph in the representative mouse. Supplementary Figure 2. The detail of identification of circRNA1615 circbase database. Supplementary Figure 3. The binding sites of circRNA1615, miR-152-3p, and LRP6. [file 8963987.f1.zip › 8963987.f1/Supplementary Figure 1.docx]

Sham group
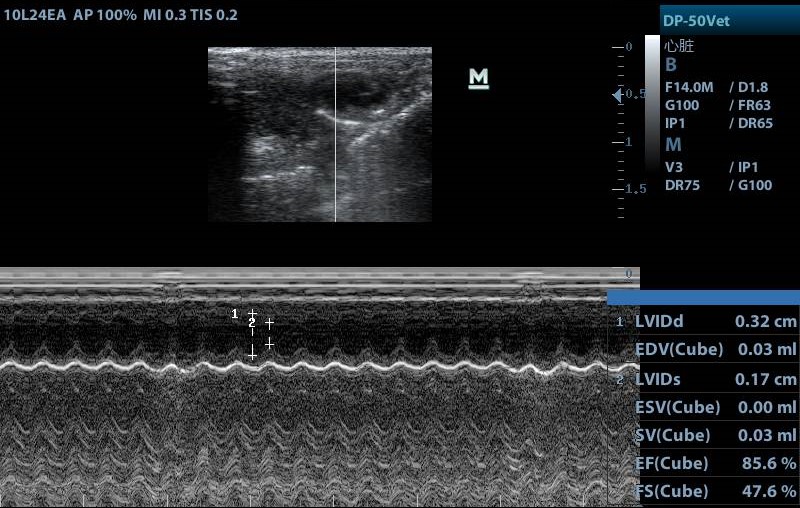


MI+NS group
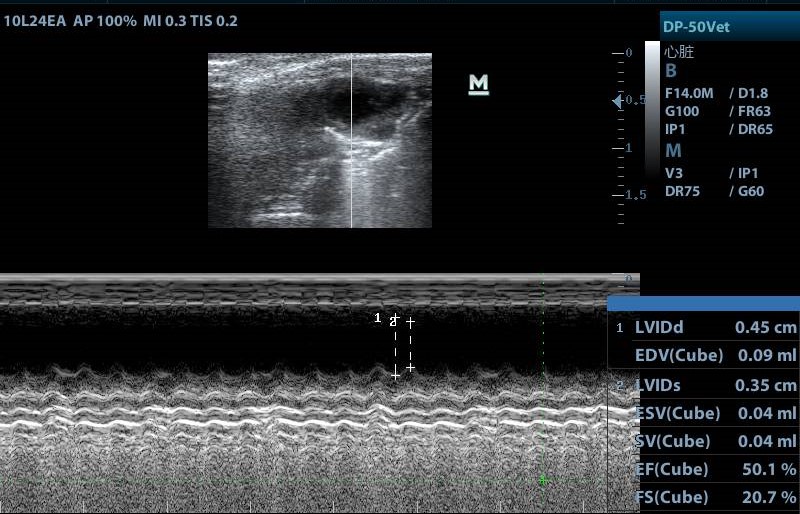


MI+Fer-1 group
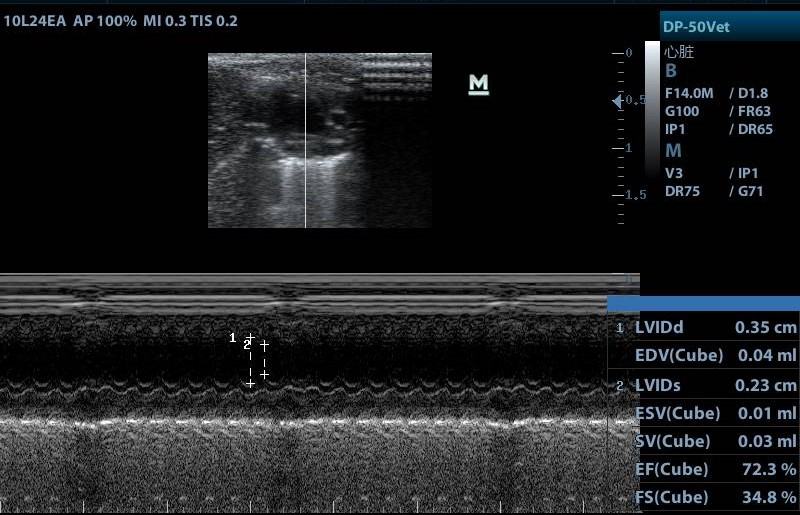


**Supplementary Figure 1. The measurement of ultrasonograph** **in the representative mouse.**

Note: NS, saline; MI, myocardial infarction; Fer-1, ferrostatin-1.
