# Supplementary material for: Effect and Mechanism of LRP6 on Cardiac Myocyte Ferroptosis in Myocardial Infarction
Source: Oxid Med Cell Longev. 2021 Oct 19;2021:8963987. doi: 10.1155/2021/8963987 (PMC8548150; doi:10.1155/2021/8963987)
Supplement: Supplementary Materials — Supplementary Figure 1 The measurement of ultrasonograph in the representative mouse. Supplementary Figure 2. The detail of identification of circRNA1615 circbase database. Supplementary Figure 3. The binding sites of circRNA1615, miR-152-3p, and LRP6. [file 8963987.f1.zip › 8963987.f1/Supplementary Figure 3.docx]

1. the binding sites of circRNA1615 wt, circRNA1615 mut and miR-152-3p


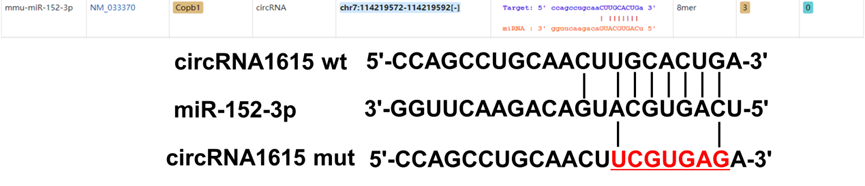


1. the binding sites of miR-152-3p and LRP6

target 5' A CCGCUAUG C UGGG C 3' (not matching bases)

LRP6 CCGGG UCUGUCA UGU GCUGG (matching bases)

miRNA GGUUC AGACAGU ACG UGACU (matching bases)

miRNA 3'A 5' (not matching bases)

**Supplementary Figure 3. The binding sites of miR-152-3p and LRP6.**

Note: Unmatched bases were inserted in the source sequence blank. target: LRP6, length: 4310; miRNA : mmu-miR-152-3p, length: 21; mfe: -24.0 kcal/mol, position 1424.
